# Xanthogranulomatous Cholecystitis Mimicking Carcinoma Gallbladder

**DOI:** 10.1155/2023/2507130

**Published:** 2023-02-13

**Authors:** Elisha Poddar, Prakash Mainali, Suraj Shrestha, Pratima Gautam, Anuradha Twayana, Niharika Pathak, Ashish Tiwari, Abhishek Bhattarai, Laligen Awale, Prasan Singh Kansakar

**Affiliations:** ^1^Maharajgunj Medical Campus, Institute of Medicine, Kathmandu, Nepal; ^2^Department of Surgery, Tribhuvan University Teaching Hospital, Kathmandu, Nepal; ^3^Department of Surgery, Patan Academy of Health Sciences, Lalitpur, Nepal; ^4^Kathmandu University School of Medical Sciences, Dhulikhel, Nepal

## Abstract

Xanthogranulomatous cholecystitis (XGC) is a rare benign chronic inflammatory disease of the gallbladder that often presents as cholecystitis and can mimic gallbladder carcinoma. Distinguishing XGC from gallbladder cancer preoperatively is challenging. We present a case of a 62-year-old male who presented with features of carcinoma gallbladder in the CECT abdomen and MRCP. Intraoperatively, there was a mass in the gallbladder and extension into the adjacent structures with involvement of the hepatic artery, 1^st^ part of the duodenum, portal vein, and hepatic flexure of the colon, and thus a palliative cholecystectomy was done. The histopathological report came out as XCG. The case aims to outline the clinical presentation of XGC and differentiate it from carcinoma gallbladder.

## 1. Introduction

Xanthogranulomatous cholecystitis (XGC) is a rare inflammatory disease of the gallbladder (GB) characterized by abnormal wall thickening and severe proliferative fibrosis with the formation of multiple yellow-brown intramural nodules [1, 2]. XGC initiates due to acute or chronic cholelithiasis, often secondary to obstruction of the bile duct, which causes the rise in intrabiliary pressure, which can cause a xanthogranulomatous inflammatory reaction after the rupture of the Rokitansky–Aschoff sinuses with extravasation of bile [3, 4]. This inflammatory process is often extensive and may extend to adjacent structures like the liver, duodenum, transverse colon, and omentum mimicking malignancy [4].

However, considering the numerous overlapping features between the XGC and gallbladder carcinoma, accurate diagnosis is difficult resulting in extended radical surgery for the XGC [1, 5]. Although various invasive and noninvasive imaging techniques have been reported, the definitive diagnosis depends on the histological examination, especially in patients with severe proliferative fibrosis involving the GB and surrounding organs [6–10].

Although XGC is not an exceptional finding, direct involvement of extra-gallbladder organs/multivisceral structures like the colon, duodenum, and right hepatic artery is very rare, with only a few cases reported in the literature [11–14].

Herein, we report a case of XGC presenting as a locally advanced gallbladder carcinoma, review the characteristics of patients with XGC involving adjacent organs, and discuss the different diagnostic and surgical options in cases of massive extra-gallbladder involvement.

## 2. Case Report

A 62-year-old man presented to our emergency department with the complaint of nonradiating right upper quadrant pain on and off for two months associated with progressive yellowish discoloration of the eye and urine for one month along with pruritus. However, the patient was not passing a pale-colored stool. The patient was febrile throughout the illness. He had 3 episodes of nonbilious vomiting containing food particles 2 days before the presentation. There was no history of anorexia or weight loss. The patient's medical, surgical history and family histories of malignancy were unremarkable.

On examination, the patient was icteric with multiple excoriations marks present all over his body. The abdominal examination was unremarkable except for mild tenderness over the right upper quadrant. Other system examination findings were normal. Admission laboratory values demonstrated deranged liver function with raised bilirubin (total-11.2 mg/dl), alkaline phosphatase (ALP) value of 262, and normal tumor markers including carcinoembryonic antigen (CEA) and alpha-fetoprotein.

**Figure 1:**
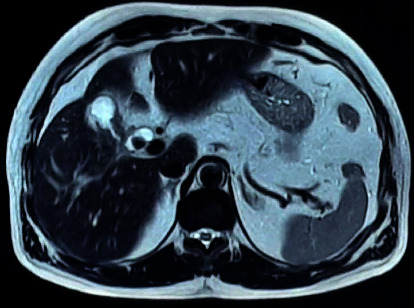
Magnetic resonance cholangiopancreatography (MRCP) showing thickened gallbladder.

**Figure 2:**
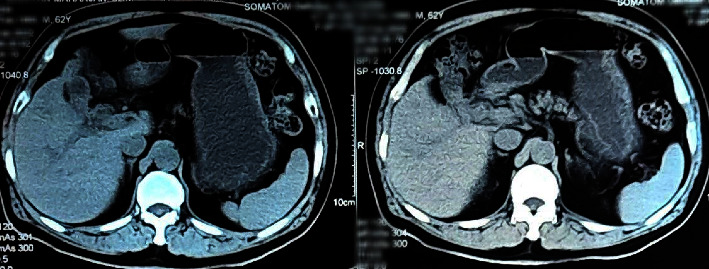
Contrast-enhanced computed tomography (CECT) abdomen pelvis showing partially distended gallbladder with thickening at the region of the body with luminal narrowing.

Magnetic resonance cholangiopancreatography (MRCP) was done, which revealed circumferential wall thickening of the body of the GB with its luminal narrowing and mild pericholecystic fat stranding suspicious of carcinoma GB along with cholelithiasis and choledocholithiasis with mild dilation of the common bile duct (CBD) (Figure 1). For further evaluation, a contrast-enhanced computed tomography (CECT) scan of the abdomen was done, which revealed a partially distended gallbladder with thickening in the region of the body resulting in luminal narrowing with calculus in the neck of the GB. Multiple fat planes were seen in the liver, hepatic flexure, and proximal duodenum with focal asymmetric thickening of the duodenal wall at the same level; features suggestive of carcinoma GB (Figure 2).

**Figure 3:**
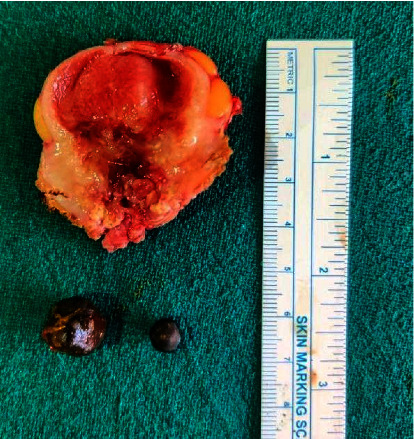
Cut section of gallbladder with stone within it.

**Figure 4:**
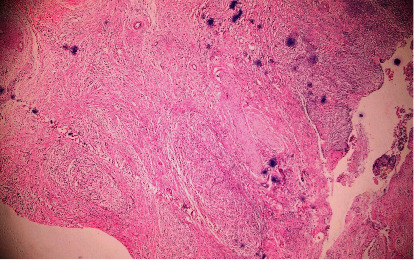
Histopathological examination of gallbladder wall infiltrated by histiocytes along with plasma cells and lymphocytes with multinucleated giant cells; features consistent with XCG cholecystitis.

After all investigations, a diagnosis of carcinoma GB with cholelithiasis with choledocholithiasis with type I Mirizzi syndrome was made. The patient was planned for open extended cholecystectomy with T-tube placement. Intraoperatively, there was a thickened GB mass extending to the liver (segments IVb and V), duodenum (D2), transverse colon, right hepatic artery, and portal vein with enlarged lymph node along the hepatoduodenal ligament. A single stone was found in the neck of GB compressing the common hepatic duct (CHD) and a CBD stone. Due to impending GB perforation and choledocholithiasis, the patient underwent open cholecystectomy with CBD exploration with stone extraction and T-tube placement (Figure 3). GB along with the mass infiltrating part of the involved organs was resected as a part of palliative surgery as there was the involvement of the right hepatic artery, hepatic flexure, and 1^st^ part of the duodenum. The postoperative period was uneventful and the patient was discharged after 10 days of hospital stay. The T-tube was removed after 3 weeks of surgery. The resected pathological specimens were sent for histopathological examination. During follow-up, histopathological report was evaluated and to our surprise, features of XGC were found showing the GB wall infiltrated by inflammatory cells composed predominantly of sheets of histiocytes along with lymphocytes and plasma cells with few multinucleated giant cells. Sections from excised lymph nodes showed intact capsular and lymphoid architecture with no granulomas and atypical cells suggesting reactive lymph nodes, all features confirming xanthogranulomatous cholecystitis (Figure 4). Nine months after surgery, the patient is asymptomatic and in good health.

## 3. Discussion

XGC is found in approximately 1.4–6% of cholecystectomies, has equal sex predilection, and is almost always accompanied by an underlying cholelithiasis (91%–100%) [15, 16]. As the inflammatory process in XGC is often extensive and can progress to involve adjacent abdominal organs, such as the liver and transverse colon that can mimic malignancy, it is crucial to differentiate XGC from advanced carcinoma GB, however, extremely difficult as in our case [17]. The involvement of surrounding viscera indicates that XGC develops aggressively similar to the advanced carcinoma GB. The presenting signs and symptoms are usually of no help differentiating these two conditions, except in advanced cases of malignancy that present with weight loss or features of ascites or metastases. None of these features were present in our case. In a study conducted by Chang et al., 25 patients with XGC and 56 patients with the wall-thickening type of T1-and T2-stages carcinoma GB, the diagnosis of whom were pathologically confirmed, the clinical symptoms, laboratory findings, and CT findings were compared. Abdominal pain, fever, and jaundice were noted more frequently in the patients with XGC. Serum aspartate aminotransferase and alanine aminotransferase levels were more elevated in patients with XGC, whereas carbohydrate antigen (CA 19-9) was higher in patients with carcinoma GB. When the T-category cancer staging of XGC and early-stage carcinoma GB were compared, diffuse GB wall thickening, intramural hypoattenuating nodule, and pericholecystic infiltration were consistent significant findings associated with XGC, regardless of the cancer staging [9]. In addition, Gallstones are more frequent in patients with XGC than in patients with carcinoma GB [18]. In the study by Feng et al., 32 of the 100 (32%) patients with XGC had choledocholithiasis [19]. The study by Kansakar et al. showed the prevalence of choledocholithiasis only among 2 of the 33 (6.06%) XGC patients [20]. Our patient had a gallstone as well as a CBD stone. GB carcinoma can co-exist in 2–15% of patients with XGC suggesting a causal association. In addition, a recent case report demonstrated that XGC showed FDG uptake on positron emission tomography, mimicking GB cancer [21].

A thorough check of the mucosa along with a frozen section examination, especially of the areas that are highly suspicious of GB cancer, can help differentiate XGC from GB cancer and help exclude the simultaneous presence of XGC and GB cancer with more accuracy [21]. Though cases of GB cancer with intraluminal polypoid masses or advanced diseases that invade adjacent organs and vessels are easy to differentiate from XGC, it is often troublesome to differentiate the wall-thickening type of early-stage GB carcinoma from XGC. On the other hand, in cases showing extensive invasion of extra-gallbladder organs, the surgical strategy should not be determined only by a frozen section examination, since it can give false negative results [13, 22].

Therefore, even if a preoperative diagnosis is made with fine-needle aspiration cytology, it is important to be aware of the possible coexistence of XGC and cancer in the same patient [23]. An incorrect diagnosis, which may be as high as 25%, results in inappropriate surgery in the form of open exploration or gallbladder bed resection for GB carcinoma rather than a simple laparoscopic cholecystectomy for XGC [16, 24]. In our reported case, the patient exhibited severe, destructive, tumor-like xanthogranulomatous inflammation, with an extensive invasion of adjacent organs and the hepatic artery making extensive surgery difficult. The clinical and radiological findings were suggestive of gallbladder carcinoma. The most probable diagnosis made was a locally advanced carcinoma gallbladder but pathological examination unexpectedly revealed an XGC. Pseudotumoral XGC has puzzled surgeons in terms of surgical treatment. Even intraoperative differential diagnosis of XGC from GB cancer remains a challenge when XGC is associated with tumor formation and adhesions to adjacent organs. As carcinoma gallbladder and XGC may coexist, radical resection, such as liver resection, is justified when malignancy cannot be completely excluded.

## 4. Conclusion

Extensive involvement of multiple viscera such as the liver, transverse colon, hepatic artery, and portal vein in XGC is very uncommon. These features often mimic carcinoma of the gallbladder, which can only be differentiated after the histological examination. In addition, an association of choledocholithiasis with XGC is rare. Radical resection should often be tried whenever possible if doubt exists regarding the underlying GB carcinoma.

## Data Availability

All the data are available within the case report.
